# Deuterated Arachidonic Acids Library for Regulation of Inflammation and Controlled Synthesis of Eicosanoids: An In Vitro Study

**DOI:** 10.3390/molecules23123331

**Published:** 2018-12-15

**Authors:** Dmitry V. Chistyakov, Ivan S. Filimonov, Nadezhda V. Azbukina, Sergei V. Goriainov, Viktor V. Chistyakov, Maksim A. Fomich, Andrei V. Bekish, Vadim V. Shmanai, Marina G. Sergeeva, Mikhail S. Shchepinov

**Affiliations:** 1 Belozersky Institute of Physico-Chemical Biology, Moscow State University, 119992 Moscow, Russia; mg.sergeeva@gmail.com; 2 All-Russian Research Institute for Optophysical Measurements (VNIIOFI), Ozernaya 46, 119361 Moscow, Russia; fis82@yandex.ru; 3 Faculty of Bioengineering and Bioinformatics, Moscow Lomonosov State University, 119234 Moscow, Russia; ridernadya@gmail.com; 4 SREC PFUR, Peoples’ Friendship University of Russia (RUDN University), 117198 Moscow, Russia; goryainovs@list.ru (S.V.G.); chistvic@gmail.com (V.V.C.); 5 Institute of Physical Organic Chemistry, National Academy of Sciences of Belarus, Surganova Str 13, 220072 Minsk, Belarus; mfomich@gmail.com (M.A.F.); andreibekish@yahoo.com (A.V.B.); v.shmanai@gmail.com (V.V.S.); 6 Retrotope, Incorporated, 4300 El Camino Real, Suite 201, Los Altos, CA 94022, USA

**Keywords:** human 15-lipoxygenase-2, human 5-lipoxygenase, human cyclooxygenase 2, deuterated arachidonic acids, eicosanoids, isotope effect

## Abstract

The synthesis of signal lipids, including eicosanoids, is not fully understood, although it is key to the modulation of various inflammatory states. Recently, isotopologues of essential polyunsaturated fatty acids (PUFAs) deuterated at bis-allylic positions (D-PUFAs) have been proposed as inhibitors of non-enzymatic lipid peroxidation (LPO) in various disease models. Arachidonic acid (AA, 20:4 n-6) is the main precursor to several classes of eicosanoids, which are produced by cyclooxygenases (COX) and lipoxygenases (LOX). In this study we analyzed the relative activity of human recombinant enzymes COX-2, 5-LOX, and 15-LOX-2 using a library of arachidonic acids variably deuterated at the bis-allylic (C7, C10, and C13) positions. Kinetic parameters (KM, V_max_) and isotope effects calculated from kH/kD for seven deuterated arachidonic acid derivatives were obtained. Spectroscopic methods have shown that deuteration at the 13th position dramatically affects the kinetic parameters of COX-2 and 15-LOX-2. The activity of 5-LOX was evaluated by measuring hydroxyeicosatetraenoic acids (8-HETE and 5-HETE) using ultra-performance liquid chromatography-tandem mass spectrometry (UPLC-MS/MS). Deuteration at the seventh and 10th positions affects the performance of the 5-LOX enzyme. A flowchart is proposed suggesting how to modulate the synthesis of selected eicosanoids using the library of deuterated isotopologues to potentially fine-tune various inflammation stages.

## 1. Introduction

The deuteration of essential polyunsaturated fatty acids (PUFAs) at bis-allylic positions was proposed as a way of controlling metabolic pathways [1]. This method was used to downregulate non-enzymatic lipid peroxidation (LPO) in various disease models [2,3,4]. An orally dosed di-deutero synthetic isotopologue of linoleic acid ethyl ester is currently being tested in human clinical trials for various diseases.

Although the clinical studies are carried out with deuterated analogues of linoleic acid, the use of deuterated arachidonic acid (AA) is attractive because AA is incorporated into membrane phospholipids, released by phospholipase A2 after inflammatory stimulation, and then metabolized into various signaling lipids (so-called eicosanoids), which act as proinflammatory or resolution mediators [5,6]. These signaling lipids belong to different families and can act individually (for example, prostaglandin E2 acts via G-protein coupled receptors [7]) or as groups, enhancing the effects of each other [8,9]. Also, these signaling moieties can be produced by transcellular biosynthesis [10]. The interest of researchers focuses on key enzymes of the AA metabolism, mainly lipoxygenase (LOX) and cyclooxygenase (COX) pathways, and a general strategy for developing their inhibitors, so-called non-steroidal anti-inflammatory drugs, has been evolving for decades [11,12]. Recently, due to significant progress in the study of the role of innate immunity in the development of inflammatory processes, alternative strategies for controlling inflammation have emerged, such as the stimulation of the resolution processes [6], polarization [13,14], and others, including the modulation, rather than inhibition, of eicosanoid synthesis [15]. Such modulation can be achieved with deuterium AA isotopologues.

Indeed, it was shown previously that bis-allylic deuteration of AA does not alter the uptake, esterification, and release of AA isotopologues from phospholipids compared to native arachidonic acid [15]. The release of eicosanoids depends on the enzymatic oxygenation of AA isotopologues. Among these enzymes, cyclooxygenase 2 (COX-2), 5-lipoxygenase (LOX-5), and 15-lipoxygenase (15-LOX-2) are the most important for inflammatory-related responses in humans [5,16]. Therefore, we tested the hypothesis that the use of various AA isotopologues may enable not only the regulation of the LPO, but also the modulation of the amounts of eicosanoids released. Accordingly, we estimated the kinetic parameters (K_M_, V_max_) of human enzymes COX-2, 15-LOX-2, and 5-LOX with AA and seven AA isotopologues deuterated at various bis-allylic positions. These data lay the framework for the regulation of eicosanoids based on the kinetic parameters obtained. Deuterated arachidonic acid derivatives can thus be utilized as a new way of soft inflammatory response modulation via the manipulation of eicosanoids synthesis. 

## 2. Results

### 2.1. COX-2 Kinetic Parameters for AA Isotopologues

**Figure 1:**
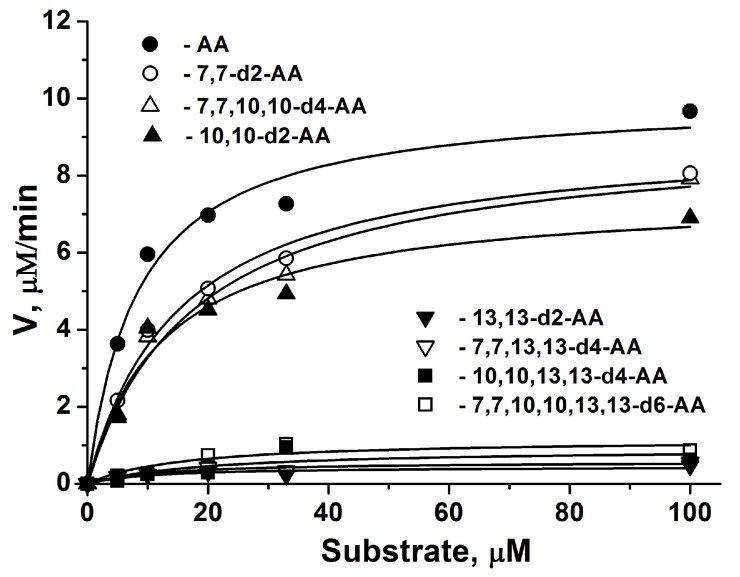
Steady state kinetics of COX-2 with arachidonic acid (AA) and deuterated arachidonic acids. Details are described in Experimental Procedures. Experiments were conducted in triplicate. Standard deviations are indicated in Table 1.

COX-2 is an important inflammatory marker, induced under inflammatory stimulation and thus a target for many anti-inflammatory drugs which act as inhibitors of the enzyme [5,17]. COX-2 catalyzes a two-step reaction (cyclooxygenase and peroxidase activities), where arachidonic acid (AA) is converted into prostaglandin H2 (PGH_2_), the precursor to several other prostaglandins, thromboxanes, and prostacyclins [17]. Thus, the enzyme activity determines the synthesis of a large number of physiologically active compounds. To assess the site of the deuteration–enzymatic activity relationship, we used the spectrophotometric method with N,N,N′,N′-tetramethyl phenylenediamine (TMPD) as the reducing co-substrate (see the Materials and Methods section). The dependence of the cyclooxygenase reaction rate on the overall AA concentration (5–100 μM) in the reaction mixture was examined (Figure 1). By fitting the Michaelis–Menten rate equation to the data, the parameters of enzyme reactions were obtained (Table 1). We observed similar K_M_ values for all AA isotopologues (Table 1). The replacement of hydrogen by deuterium at the 13th carbon atom resulted in a significant decrease (more than 10 times) in V_max_ for compounds 13,13-d2-AA, 7,7,13,13-d4-AA, 10,10,13,13-d4-AA, and 7,7,10,10,13,13-d6-AA (Table 1). Therefore, compounds with deuterium in the 13th position may be used to downregulate the synthesis of the COX-dependent family of eicosanoids, while if it is necessary to preserve this synthesis, compounds with deuterium in the seventh position should be used, as this isotopologue still downregulates LPO.

### 2.2. 15-LOX-2 Kinetic Parameters for AA Isotopologues

**Figure 2:**
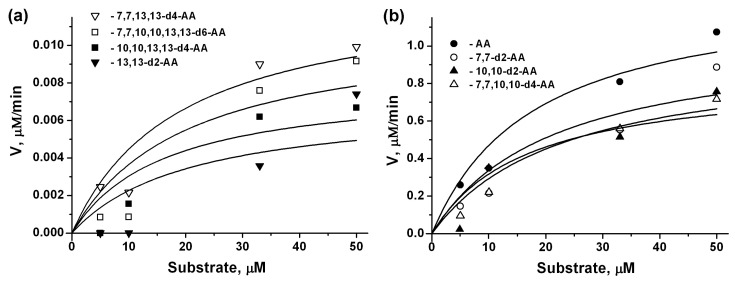
Steady state kinetics of 15-LOX-2 with arachidonic acid (AA) and deuterated arachidonic acids. (**a**) 13,13-d2-AA; 7,7,13,13-d4-AA; 10,10,13,13-d4-AA; 7,7,10,10,13,13-d6-AA; (**b**) AA, 7,7-d2-AA; 10,10-d2-AA; 7,7,10,10-d2-AA. Details are described in Experimental Procedures. All experiments were conducted in triplicate. Standard deviations are indicated in Table 1.

Arachidonate 15-lipoxygenase type II (15-LOX-2) metabolizes arachidonic acid to 15-hydroperoxyeicosatetraenoic acid (15-HpETE), which is further metabolized to 15-hydroxyeicosatetraenoic acid (15-HETE), a precursor to a family of derivatives with mainly pro-resolving properties [18]. The enzyme has a negligent ability to metabolize linoleic acid and therefore its sensitivity for various AA isotopologues is important for the synthesis of these substances [19]. To evaluate the 15-LOX-2 activity depending on the deuteration position, we used the spectrophotometric method, measuring the formation of the conjugated diene product at 234 nm. We obtained a significant kinetic parameters difference between AA isotopologues with bis-allylic deuteration at position 13 (Figure 2a) and others (Figure 2b). By fitting the Michaelis-Menten rate equation into the data, the parameters of enzyme reactions were obtained (Table 1). The K_M_ values for all AA isotopologues were similar (Table 1). The replacement of hydrogen by deuterium at position 10 (7,7,10,10-d4-AA, 10,10-d2-AA) has a small effect (Figure 2b, Table 1). Thus, to control this branch of the eicosanoids synthesis, it is necessary either to exclude deuterium in the 13th and 10th positions (potential enhancement of the synthesis of pro-resolution factors), or deuterate the said bis-allylic positions to inhibit the product formation.

### 2.3. 5-LOX Kinetic Parameters for AA Isotopologues, Estimated by 5-HETE and 8-HETE Products

**Figure 3:**
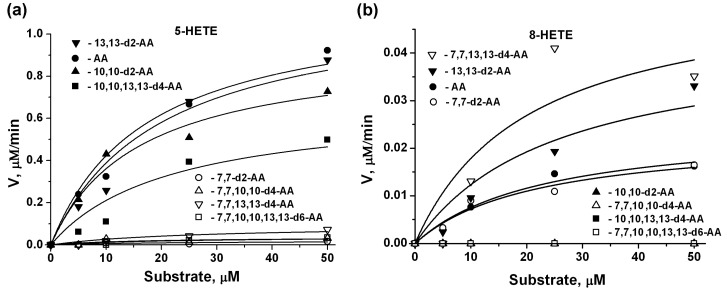
Steady state kinetics of 5-LOX with arachidonic acid (AA) and deuterated arachidonic acids monitoring the formation of 5-HETE (**a**) and 8-HETE (**b**) by using UPLC-MS/MS. Details are described in Experimental Procedures. All experiments were conducted in triplicate. Standard deviations are indicated in Table 2.

Human 5-LOX plays an important role in various diseases, ranging from asthma to cancer, and is considered a potential therapeutic target [20]. 5-Lipoxygenase (5-LOX) catalyzes the first two steps in the conversion of arachidonic acid to proinflammatory leukotrienes [21]. The reaction of 5-LOX with AA generates two products, from hydrogen abstraction at C7 (95% 5-HETE) and at C10 (5% 8-HETE) [22]. We therefore measured the activity of these enzymes by product detection with UPLS-MS/MS (see Material and Methods). To avoid the 5-LOX lag phase, we added 13(S)-HpODE (to oxidize Fe^2+^ to Fe^3+^) (Figure 3). The parameters of the reaction are presented in Table 2.

We observed that deuteration at positions 10 or 13 had little effect on the rate of 5-HETE formation. However, using tetra-substituted 10,10,13,13-AA-d4 as a substrate, V_max_ decreased almost two-fold (Table 2). Deuteration at C10 sharply decreased the 8-HETE formation rate (Figure 3). Such blocking of the 8-HETE synthesis was associated with the fact that this reaction is normally an adverse reaction and an increase in the activation barrier leads to strong inhibition. Deuteration at position C13 elicited a significant (almost two-fold) increase in the rate of formation of 8-HETE (Table 3, 13,13-d2-AA and 7,7,13,13-d4-AA).

## 3. Discussion

**Figure 4:**
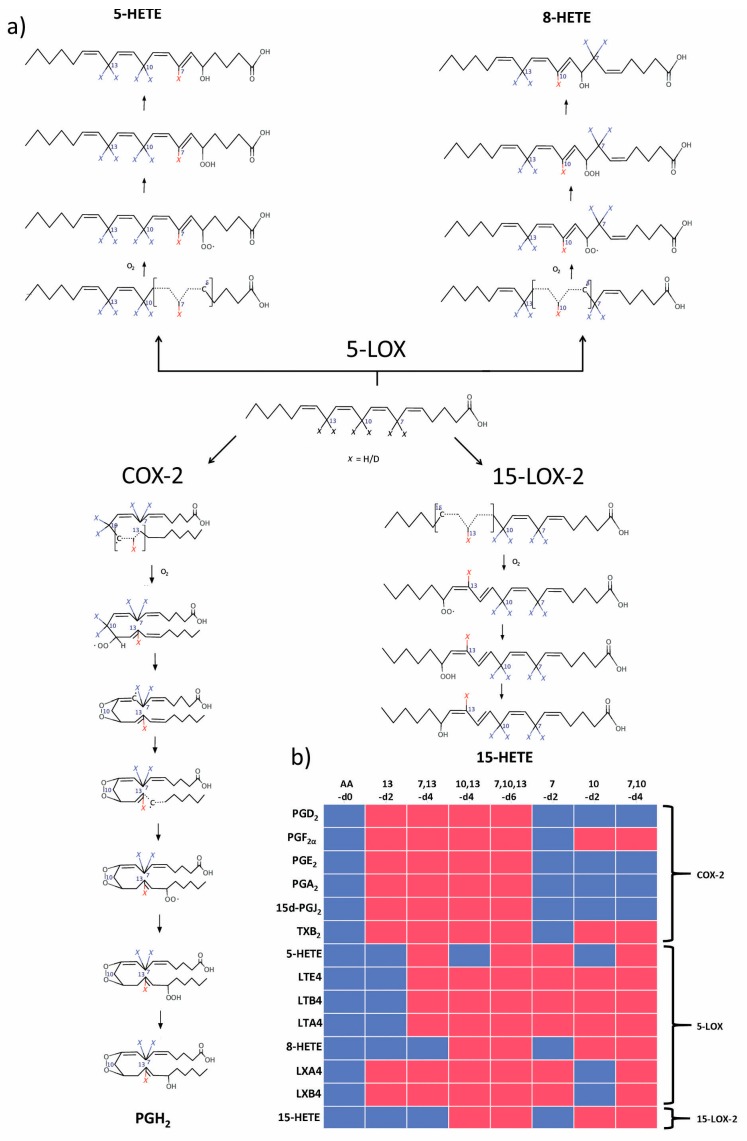
(Bis-allyl)-deuterated AA library for a controlled synthesis of eicosanoids. (**a**) Reaction mechanism scheme. (**b**) Intended effects of treatment with different deuterated AA on pro- and anti-inflammatory eicosanoids synthesis in vivo (red—reduced reaction rate, blue—no effects).

The modulation of eicosanoid synthesis is an important aim in the study of innate immunity pathologies, particularly of the inflammation processes [5,23]. Our results show that using various (bis-allyl)-deuterated AA isotopologues, one can effectively control eicosanoid synthesis. The effects of the D-AA isotopologue library on the output of the studied enzymes are shown in Figure 4.

According to the general ideas about the reaction mechanism of COX-2 [24] and our present data, deuteration at position 13 decreases the enzyme activity (Figure 4). The kinetic parameters for COX-2 activities measured for AA and 13,13-d2-AA are similar to those previously published [24,25]. It was shown that deuteration at position 10 affects the prostaglandins synthesis in macrophages [15]. Our data provide further insight on this point. We observed no influence of deuteration at this position on COX-2 activity, and accordingly suggest that this atom is important for secondary prostaglandin synthase enzymes (prostaglandin E synthases, prostaglandin D synthase, etc.). Alternatively, since cells were saturated for 24 h with 25 μM AA or its isotopologues [15], but since it is known that concentrations of 10 μM and above could be identified as proinflammatory stimuli which induce cellular responses [26], some other mechanisms depending on deuterium at position 10 could not therefore be excluded and require further consideration. 

The mechanism of 15-LOX-2 has been studied in detail [27,28] since the role of this enzyme as a source of proinflammatory eicosanoids and related substances became clear [29,30]. Suggested mechanisms are summarized in Figure 4. We assign the key role to position 13, since all AA isotopologues with deuterium at this position demonstrate a low reaction rate and high kinetic isotope effect KIE (Table 1). The fact that deuteration at position 13 inhibits 15-LOX-1 [31] suggests that it operates through a mechanism similar to that for 15-LOX-2 [29]. 

5-LOX and 15-LOX-2 belong to a family of human lipoxygenases. The mechanisms of formation of 5-HETE and 8-HETE are described [22] and summarized in Figure 4. The difference between 5-HETE and 8-HETE formation changes the ratio between these eicosanoids with the 7,7-d2-AA and 7,7,13,13-d4-AA isotopologues. 

The activities of the tested enzymes are instrumental in the synthesis of eicosanoids and related compounds [5]. Taken together with these previously described metabolic branches [5], our data suggest the following scheme for using deuterated AA isotopologues to modulate eicosanoid production (Figure 4b). AA undergoes enzymatic transformation through multiple pathways and the inhibition of some is not always good for the system as a whole. Nonetheless, one can see that using various isotopologues makes it possible to downregulate the synthesis of one family of signaling lipids while upregulating others (red and blue fields in Figure 4b), e.g., modulating the synthesis to a good effect.

An important factor for pharmacological intervention using deuterated AA is the dilution by adipose triglyceride PUFA stores and dietary intake of AA and its precursor linoleic acid. The average dietary AA uptake in the Western world is approximately 100 mg per day, exceeded by at least a hundred-fold by the uptake of linoleic acid (10–20 g per day). Pharmacokinetic data from a recent clinical Friedreich’s Ataxia study with up to 9 g per day of oral deuterated linoleic acid showed only modest levels of ca. 10% 13,13-d2-AA in plasma and up to 25% in red blood cells after 28 days [32]. While these levels have been proven to suppress non-enzymatic lipid peroxidation, which has exponential kinetics [1,2,3,4], it is challenging to modulate enzymatic oxidation significantly when the deuterated PUFA is present in sub-stoichiometric amounts. However, dilution is much less relevant for topical applications, which are in widespread use to treat inflammatory skin conditions.

In summary, we propose a potential application of pharmacological agents based on deuterated fatty acids as possible regulators of eicosanoid synthesis. We investigated the mechanisms of interaction of human enzymes of the arachidonic acid cascade with arachidonic acids deuterated at various positions. Kinetic parameters (K_M_, V_max_) for seven deuterated arachidonic acid derivatives were obtained. We showed that deuteration in the 13th position dramatically affects the kinetic parameters of the COX-2 and 15-LOX-2 enzymes, and deuteration in the seventh and 10th positions affects the operation of the 5-LOX enzyme, which opens up the possibility of controlling the synthesis of eicosanoids. Although the use of selected labeled arachidonic acid analogues as mechanistic probes for enzymes has been proposed [33], only recently have the deuterated analogues of polyunsaturated fatty acids been effectively applied in cell models and in animals [1,2,3,4]. Taken together with our present data, these findings suggest the polyunsaturated fatty acids deuterated analogues library to be a new promising way for controlling inflammatory processes. 

## 4. Materials and Methods

### 4.1. Chemicals 

All chemicals were of the highest purity available and obtained from commercial sources. Tris base (2-amino-2-(hydroxymethyl)-1,3-propanediol), potassium hexacyanoferrate (II) trihydrate (ferrocyanide), arachidonic acid, phosphatidylcholine, sodium hydroxide, and sodium chloride were obtained from Sigma Aldrich (St. Louis, MO, USA). Tween-20, TMPD (N,N,N′,N′-tetramethyl-p-phenylenediamine were obtained from (MP Biomedicals, Solon, OH, USA); ATP was obtained from (Fermentas, Vilnius, Lithuania); and 13(S)-HpODE, polysorbate 20, 12-HETE-d8, and 5-HETE-d8 were obtained from (Cayman Chemicals, Ann Arbor, Michigan). All the other chemicals were obtained from standard sources.

### 4.2. Arachidonic Acid Library

The synthesis and analytical verification is described elsewhere [34]. The isotopologues of arachidonic acid (AA) used in this study were 7,7-(D2)-AA, 10,10-(D2)-AA, 13,13-(D2)-AA, 7,7,10,10-(D4)-AA, 7,7,13,13-(D4)-AA, 10,10,13,13-(D2)-AA, and 7,7,10,10,13,13-(D6)-AA. These AA compounds were synthesized as ethyl esters for improved stability during storage. AA-ethyl esters were hydrolyzed to free acid by base hydrolysis for experiments. Briefly, a solution of AA-ethyl ester in methanol (3 mg/mL) was hydrolyzed with 0.5 M KOH in a round-bottom flask and capped under argon gas for 2 h at 70 °C. The reaction solution was then diluted by 1/3 with HPLC-grade water, and then acidified with 160 mM H_2_SO_4_. The solution was then extracted with hexane three times. The organic phase was transferred to a borosilicate tube and evaporated under a flow of argon. The final residue was dissolved in 1 mL of ethanol and stored at −80 °C. 

### 4.3. COX-2 Kinetic Assays

The peroxidase activity of human recombinant COX-2 (Biomole, Hamburg, Germany) was measured spectrophotometrically by using 170 μM N,N,N′,N′-tetramethyl phenylenediamine (TMPD) (Sigma-Aldrich), as the reducing co-substrate over a 5-min period [35]. Total COX-2 activity was determined using the TMPD extinction coefficient (0.00826 μM^−1^), and control wells containing no COX-2 were subtracted as background non-enzymatic oxidation. Stock solutions of TMPD were prepared prior to experiments. The stock vial with the enzyme was maintained on ice (0–4 °C) at all times when performing experiments. Other stock solutions were stored at −20 °C. Buffer solution (50 mM Tris HCl, pH 8.0, tween-20 0.1%, heme 1 mM, EDTA 5 mM) was stored at 4 °C. Peroxidase reaction was monitored spectrophotometrically at 590 nm by the accumulation of the oxidized form of TMPD. Absorbance was measured with a Synergy H4 plate reader (BioTek, Winooski, VT, USA) in a 96-well plate. 

The assay reaction mixture volume was 220 μL. Buffer solution with additions of required tween-20 overall content and aliquots of TMPD and hydrogen peroxide stock solutions were added to the cell. Reaction was initiated by enzyme addition to the reaction mixture. TMPD, arachidonic acids, and COX-2 aliquots were added with Hamilton micro syringes. Reaction temperatures were maintained at 25 °C using the spectrophotometer temperature control system.

### 4.4. 5-LOX Kinetic Assays

Human recombinant 5-LOX (Biomole, Hamburg, Germany) activity was measured using the UPLC-MS/MS method. For each 5-LOX run, the substrate (AA, 7,7-d2-AA, 10,10-AA-d2, 13,13-AA-d2, 7,7,10,10-AA-d4, 7,7,13,13-AA-d4, 10,10,13,13-AA-d4, 7,7,10,10,13,13-AA-d6; 5–40 μM) was combined with the enzyme (5-LOX; 6.7 U) in 110 μL of Tris buffer (50 mM, pH 7.5, 25 °C, 2 mM CaCl_2_, 1 mM ATP, 25 μg/mL phosphatidylcholine). The stock vial with the enzyme was maintained on ice (0–4 °C) at all times when performing experiments. The activator, 13(S)-HPODE, was added to the buffer to a final concentration of 2.5 μM. The substrate and the activator were first combined in the assay buffer. The reaction was terminated after 2 min by the addition of 100 μL of MeOH to the total reaction mix. 

Lipids were extracted from reaction samples using the solid-phase extraction method. For solid-phase extraction, 1 mL hydrophilic-lipophilic balance (HLB) (Oasis^®^ HLB cartridge (60 mg, 3cc) cartridges were washed with 1 mL of methanol and 1 mL of 0.1% formic acid. Samples were loaded onto the column and washed with 1 mL 0.1% formic acid and 1 mL 15% ethanol. The cartridges were then eluted with 300 μL methanol. 5-HETE and 8-HETE were analyzed by 8040 series UPLC-MS/MS (Shimadzu, Kyoto, Japan) with all specifications set as previously reported [8]. Quantification and qualification were accomplished in multiple-reaction monitoring mode, and MS was operated at unit mass resolution for both precursor and product ions. See Table 1 for a list of the multiple-reaction monitoring (MRM) pairs used to quantify 5-HETE and 8-HETE derived from different deuterated arachidonic acids.

Lipid mediator version 2 software package (Shimadzu, Kyoto, Japan) was used to operate the mass spectrometer (Shimadzu). Lipid mediators were separated based on their chemical properties in UPLC, then we monitored their ion fragments by collision-induced dissociation in conjunction with electrospray ionization-MS/MS. 5-HETE and 8-HETE were identified according to accurate *m*/*z*, retention time, relative retention time of species in the same class, and spectra of MS/MS.

### 4.5. 15-LOX-2 Kinetic Assays

15-Lipoxygenase-2 human recombinant (Biomole, Hamburg, Germany) reaction rates were determined by following the formation of the conjugated diene product at 234 nm (ε = 25,000 M^−1^ cm^−1^) with a Synergy H4 plate reader (BioTek, Winooski, VT, USA) in a 96-well plate. For each 15-LOX-2 run, a substrate was combined with the enzyme (15-LOX-2; 0.5 U/reaction) in 200 uL of Tris buffer (50 mM, pH 7.2, 37 °C, containing 0.003% polysorbate 20). The stock vial with the enzyme was maintained on ice (0–4 °C) at all times when performing experiments. Mathematical processing of all experimental data was carried out using the Origin 7.5 package from MicroCAL Software (Microcal Software, Inc., Northampton, MA, USA).

## Figures and Tables

**Table 1:** Kinetic parameters for the reaction(s) of COX-2 and 15-LOX-2 with AA and deuterated AA isotopologues.

	COX-2 *			15-LOX-2 **		
AA Isotopologues	V_max_ (µM/min)	K_M_ (µM)	Isotope Effect Calculated from H_kcat_/D_kcat_	V_max_ (µM/min)	K_M_ (µM)	Isotope Effect Calculated from H_kcat_/D_kcat_
AA	11.07 (1.1) ***	10.8 (4.1)		1.316 (0.099)	18.0 (3.6)	
7,7-d2-AA	9.11 (0.7)	15.4 (3.2)	1	1.056 (0.088)	21.4 (4.3)	1
10,10-d2-AA	7.53 (0.41)	12.7 (2.8)	1	0.973 (0.077)	23.2 (4.6)	1
13,13-d2-AA	0.44 (0.21)	10.7 (3.5)	25	0.007 (0.001)	20.9 (4.2)	188
7,7,10,10-d4-AA	8.61 (0.82)	15.8 (2.9)	1	0.844 (0.063)	16.6 (3.3)	2
7,7,13,13-d4-AA	0.58 (0.25)	12.2 (3.1)	19	0.013 (0.001)	19.1 (3.8)	101
10,10,13,13-d4-AA	0.83 (0.22)	17.6 (3.7)	13	0.008 (0.001)	16.4 (3.3)	165
7,7,10,10,13,13-d6-AA	1.07 (0.31)	13.8 (3.6)	10	0.011 (0.001)	20.1 (4.0)	120

* Kinetic parameters for the rates of PGH2 formation by COX-2 were monitored spectrophotometrically at 590 nm (accumulation of the oxidized form of TMPD). ** Kinetic parameters for the rates of 15-HPETE formation by 15-LOX-2 were monitored following the formation of the conjugated diene product at 234 nm. *** Standard deviations are indicated in parentheses.

**Table 2:** Kinetic parameters for the rates of 5-HETE and 8-HETE formation by 5-LOX with AA and deuterated AA using UPLC-MS/MS. Standard deviations are indicated in parentheses.

	5-HETE			8-HETE			Product Ratio
	V_max_ (µM/min)	K_M_ (µM)	Isotope Effect Calculated from Hkcat/Dkcat.	V_max_ (µM/min)	K_M_ (µM)	Isotope Effect Calculated from Hkcat/Dkcat.	(5-HETE/8-HETE),%
AA	1.151 (0.21)	17.3 (3.5)	-	0.025 (0.008)	22.9 (4.6)	-	98/2
7,7-d2-AA	0.020 (0.01)	22.4 (4.5)	58	0.023 (0.007)	21.7 (4.3)	1	51/49
10,10-d2-AA	0.933 (0.15)	15.9 (3.2)	1	n/a	n/a	-	100/0
13,13-d2-AA	1.154 (0.12)	19.8 (4.0)	1	0.043 (0.004)	25.2 (5.0)	0.5	98/2
7,7,10,10-d4-AA	0.037 (0.015)	16.6 (3.3)	31	n/a	n/a	-	100/0
7,7,13,13-d4-AA	0.093 (0.015)	25.6 (5.1)	12	0.054 (0.015)	20.4 (4.1)	0.5	60/40
10,10,13,13-d4-AA	0.665 (0.09)	21.7 (4.3)	2	n/a	n/a	-	100/0
7,7,10,10,13,13-d6-AA	0.040 (0.02)	23.5 (4.7)	29	n/a	n/a	-	100/0

**Table 3:** Multiple reaction montitoring (MRM) transitions for 5-HETE and 8-HETE derived from deuterated arachidonic acids.

Analyte	AA	7,7-AA-d2	10,10-AA-d2	13,13-AA-d2	7,7,10,10-AA-d4	7,7,13,13-AA-d4	10,10,13,13-AA-d4	7,7,10,10,13,13-AA-d6
5-HETE	319-115	320-115	321-115	321-115	322-115	322-115	322-115	324-115
8-HETE	319-155	321-157	320-155	321-155	323-157	323-157	323-157	324-157
